# The venezuelan scientific diaspora as a transnational actor: knowledge circulation, STI cooperation, and development

**DOI:** 10.3389/frma.2026.1870192

**Published:** 2026-09-07

**Authors:** Luisa F. Echeverría-King, Branislav Pantović, Kleinsy Bonilla, Daniela Moreno Garcia, Estiven González-Sarmiento

**Affiliations:** 1 Universidad Simón Bolívar, Barranquilla, Colombia; 2 DiploCientífica, Viedma, Argentina; 3 Observatorio Económico Sostenible, Universidad del Valle de Guatemala, Ciudad de Guatemala, Guatemala

**Keywords:** brain linkage, knowledge circulation, Latin America and the caribbean, science diplomacy, scientific cooperation, scientific diaspora, Venezuela, migration

## Abstract

This study examines the potential of the Venezuelan scientific diaspora to contribute to the development and strengthening of scientific and technological ecosystems in their countries and regions of residence. This study examines the engagement capacities, interests, collaborative practices, and perceived barriers of the Venezuelan scientific diaspora residing across different world regions. It further considers how these transnational capacities could support STI cooperation involving Venezuela and Latin America and the Caribbean. The methodology combines qualitative and quantitative approaches. Firstly, the article draws on the literature on scientific diasporas, transnational knowledge circulation, and ecosystem resilience to analyze the scientific diaspora as a transnational actor. Secondly, an online survey broadens the empirical base by capturing the experiences and perspectives of Venezuelan scientists abroad beyond formal networks. Thirdly, participatory focus groups with representatives of the Venezuelan scientific diaspora worldwide are used to identify key activities, strategies, and mechanisms of engagement. The findings provide a comprehensive understanding of the barriers to and opportunities for international cooperation and culminate in a set of actionable recommendations to strengthen the role of the Venezuelan scientific diaspora in advancing regional scientific and technological international collaboration.

## 1 Introduction

The broader political, economic, and geopolitical context shapes not only the causes of Venezuelan scientific mobility, but also the conditions under which transnational collaboration can occur. The international mobility of highly qualified scientists has traditionally been analyzed through the perspective of “brain drain,” understood as a loss of human capital to more developed economies. Some authors argue that “talent is often wasted at home” (Agrawal et al., 2011). However, in recent decades, this view has evolved toward models that emphasize the importance of knowledge circulation, in which scientific diasporas act as transnational actors of cooperation and development in global science, technology, and innovation (STI). These dispersed researchers foster knowledge transfers, institutional collaborations, and opportunities for international cooperation, creating added value for both countries of origin and host countries (Warner et al., 2022).

Similarly, recent literature has stressed that the scientific diaspora should not be seen only as a loss of human capital, but rather as a strategic opportunity for knowledge transfer and international cooperation (Páez, 2024). In Latin America, the scientific diaspora has emerged as a strategic resource for developing scientific ecosystems, where global connectivity, mediated by science internationalization and science diplomacy, enables diaspora communities to overcome local research constraints and create strong scientific ties. The role of the diaspora is seen not only as a facilitator of technology transfer but also as a bridge to public policies that promote cooperation and sustainable technological development (Echeverría-King L et al., 2022). Particularly, the Venezuelan scientific diaspora is a phenomenon of great importance for skilled migration. It is a case of scientific mobility under crisis, institutional fragility, weakened STI infrastructure, and regional displacement. That makes the case more analytically interesting than diaspora engagement in relatively stable scientific ecosystems.

Since the beginning of the 21st century, the Venezuelan case has been characterized by a sustained outflow of highly trained professionals in the fields of STI, driven by structural factors related to the economic crisis, political instability, and precarious working conditions in the academic and scientific sectors (Scharifker, 2017). Immediate impacts include both the economic contribution of remittances and a critical loss of human capital in sectors essential for national recovery, such as education, health, and oil.

The national science and technology system has suffered the effects of this process (Mercado et al., 2025), but it has also given rise to a transnational network of scientists who, despite the physical distance, maintain ties with their country of origin. Nowadays, the scientific diaspora from Venezuela is both diverse across disciplines and is integrated into leading universities and research centers in the world (Páez, 2024), but its interaction with Venezuela is hindered by to the lack of adequate public policies, institutional weakness, and limitations of the national scientific infrastructure. The gap between strong diasporic identification and weak formal organization does not imply absence of transnational activity; rather, it suggests that many forms of diaspora engagement operate through dispersed, informal, and unevenly institutionalized cross-border networks (Portes et al., 1999).

The potential contribution of the Venezuelan scientific diaspora can also be understood through the lens of social remittances. Beyond physical return, diaspora scientists transfer ideas, skills, professional practices, research norms, and institutional know-how through mentoring, teaching, collaboration, and advisory roles (Levitt, 1998; Agrawal et al., 2011). This article suggests that these ideas and practices can be applied in both host and home countries, where they may help transform social and political life.

However, the potential of the Venezuelan scientific diaspora remains only partially recognized. A persistent tension runs through the narratives of its members: on the one hand, a strong willingness to collaborate and contribute to Venezuela's development; on the other, a marked distrust toward institutional continuity, bureaucratic constraints, and the broader political environment. This tension is not merely rhetorical—rather, it shapes concrete forms of engagement, as collaboration is envisioned through specific practices such as research partnerships, teaching, mentoring, knowledge transfer, consulting, and institutional strengthening. Yet, these efforts are often hindered by the absence of adequate public policies, institutional fragility, and limitations in the national scientific infrastructure (Pantović et al., 2026).

Against this backdrop, the study examines the Venezuelan scientific diaspora as a transnational actor whose members reside all over the world. Latin America and the Caribbean are treated as a principal policy and cooperation arena rather than as the exclusive geographical location of the study population. The analysis therefore explores how globally distributed capacities, networks, and willingness to collaborate could support Venezuela and broader regional STI cooperation. This framing builds on transnational migration theory, the concept of social remittances, and evidence that skilled diasporas can function as “brain banks” and channels of international scientific-technology diffusion (Portes et al., 1999; Levitt, 1998; Agrawal et al., 2011; Kerr, 2008).

Drawing on focus groups and survey data, the article identifies key challenges, capacities, and expectations within the diaspora, and formulates strategic recommendations for (inter)national funding agencies to strengthen its connection with the country of origin. In doing so, it underscores the critical role that this community can play in supporting the recovery and future development of Venezuela's science and technology system, particularly in a context marked by prolonged crisis and shifting geopolitical dynamics.

## 2 Context of Venezuela— characteristics and specificities

The scale of Venezuelan migration is a central, though indirect, feature of this article. UNHCR, the UN Refugee Agency, estimates that nearly 7.9 million people have left Venezuela in search of protection and a better life. The majority—more than 6.9 million people—are hosted in Latin American and Caribbean countries (UNHCR, 2025). Likewise, the World Bank characterizes Venezuelan migration among the largest human mobilization in the region's recent history (World Bank, 2024) and according to the International Organization for Migration, Venezuela has remained in a prolonged period of instability, characterized by persistent socioeconomic challenges, mounting pressure on public services, and a pervasive sense of uncertainty (IOM, 2026). Therefore, the Venezuelan case cannot be understood solely through the conventional lens of skilled emigration or “brain drain.” It must be situated within a broader process of social, political, economic, and institutional disruption that has affected higher education, research funding, academic mobility, and the country's integration into contemporary international knowledge networks.

Nevertheless, aggregate migration figures should not be mechanically equated with the scientific diaspora. Available data demonstrate the scale of Venezuelan displacement and mobility, but they do not, by themselves, reveal the size, disciplinary composition, institutional location, or collaborative practices of Venezuelan scientists and academics abroad. This is an important analytical caution. For this reason, the article required engagement with existing diaspora organizations, and qualitative approaches capable of capturing informal and transnational forms of collaboration that remain invisible in official statistics. The deterioration of scientific and academic conditions in Venezuela reinforces the importance of this diaspora.

The report on Colombian diaspora described a context in which lack of funding and restrictions on academic freedom have left researchers feeling a sense of hopelessness and pondering an “exodus” from the country (Prieto et al., 2025). In addition, various studies argue that scientific publications in Venezuela declined over a 15-year period, mainly because of emigration, and warn that without corrective action, some fields could be abandoned as research topics (Morgado et al., 2025). This can support the broader claim that the country's research capacity has been affected not only by the departure of people, but also by the erosion of institutional conditions that allow science to be practiced, funded, and internationally connected" (Scharifker, 2017).

This is also consistent with the wider literature on scientific diasporas. Highly qualified migration has been consolidated as one of the most visible features of the global economy of knowledge. Rather than being limited to the individual displacement of professionals, this process recon scientific capabilities, academic trajectories, research networks and possibilities for technological development both in countries of origin and in countries of destination. The Venezuelan case is clearly inscribed in this issue. During the last decades, Venezuela has experienced a significant emigration of scientific and highly skilled talent (Scharifker, 2017).

This is supported by an earlier IDB study indicating that Venezuela lost a substantial share of its research workforce through migration, estimating a loss of 16% in 2020 (Diez et al., 2020). Likewise, Venezuelan qualified migration has also been framed as talent flight and a loss of human capital with consequent constraints on national development (Sanchez, 2020), affecting Venezuelan science especially in engineering, health, and education. This leaves the country without a critical mass of researchers that will be difficult to restore without international cooperation (Portillo, 2019).

This reading is insufficient if one does not consider that a part of this population maintains links, collaborative sectors and articulation arrangements that do not disappear with migration, but that can transform and acquire new forms in transnational spaces. Precisely for this reason, the Venezuelan scientific diaspora constitutes an object of study associated with the loss, as well as an expression of the contemporary reconfiguration of scientific capital in contexts of international movements.

According to Diez et al. (2020), the regional dimension of the Venezuelan research diaspora became more pronounced in the last decade, since from 2014 Latin America became the main destination region for Venezuelan researchers. The authors also note that “researchers who migrated to Latin America […] are also younger than researchers currently living in North America and Europe,” (Diez et al., 2020., p. 8) suggesting that part of this diaspora may still be developing or consolidating its academic and professional trajectories. This matters for Latin America and the Caribbean because engagement with the Venezuelan scientific diaspora can support the formation of new regional careers, networks, and collaborative agendas. Likewise, this might be analytically important because Venezuelan researchers based in Latin America may be embedded in scientific, linguistic, institutional, and development contexts closer to those of Venezuela. In this sense, the regional benefit also lies in creating conditions for these researchers to grow within Latin American STI ecosystems while contributing to shared capacities through joint research, mentoring, training, technology transfer, and institutional cooperation. Within this context, the Venezuelan scientific diaspora becomes relevant not only as a displaced population, but also as a distributed knowledge infrastructure and resource, and it should be analyzed as an actor in knowledge circulation.

## 3 Theoretical framework and literature

In this article the scientific diaspora is defined as a community of highly qualified professionals who have emigrated from their country of origin, might have maintained academic, social, and cultural ties with it, and developed transnational networks of cooperation. This perspective recognizes that communities of emigrated researchers can act as agents of knowledge transfer, catalyzing cooperation between institutions in different contexts and fostering cross-border exchanges of scientific skills and resources (Warner et al., 2022).

As highlighted earlier, the theoretical debate on the dynamics of highly skilled migration has shifted from the classic brain drain paradigm to more relational and less linear approaches. The notion of “brain drain” understands qualified migration as a net transfer of capabilities from countries with fewer structural opportunities to more consolidated scientific and economic systems. This perspective emphasizes the deterioration of the national scientific base, the loss of critical mass, and the difficulty of sustaining endogenous innovation processes. Nevertheless, as Docquier and Rapoport note, since the late 1990s a “third wave” of research has examined the fact that “the brain drain has both detrimental and beneficial effects for origin countries” and has sought to identify “the conditions under which the net effect on development and welfare is positive or negative” (Docquier and Rapoport, 2012; p. 3).

This tension is central to the Venezuelan case: skilled emigration may weaken local scientific networks, but it can also create a “brain bank” when researchers abroad maintain links that allow domestic actors to access knowledge, contacts, and opportunities accumulated internationally (Agrawal et al., 2011). Kerr suggests that diaspora scientists can act as bridges for knowledge circulation when their professional and scientific networks connect expertise acquired abroad with institutions and innovation needs in the country of origin. In this sense, the Venezuelan scientific diaspora may contribute not only through return, but also through cross-border channels of technology diffusion, collaboration, and STI capacity building (Kerr, 2008).

Shin and Moon argue that “there has been a significant shift in conceptualizing talent flows” from labor understood mainly as human capital toward labor understood as social capital, where networks generate “enhanced trust and cooperation, information sharing, and improved access” to development opportunities (Shin and Moon, 2018; p. 9). Within this shift, the brain circulation approach challenged the static logic of brain drain by showing that mobility can generate return flows, knowledge exchange, training, and the transfer of skills acquired abroad. However, when return is neither mass nor immediate, brain circulation remains insufficient to capture more complex and sustained forms of engagement between diasporic communities and their countries of origin. This is where the brain linkage perspective becomes especially relevant. For Shin and Moon, brain linkage refers to “home-host interactions” through which highly skilled emigrants may engage with their countries of origin “through business visits or even short-term stays, if not returning permanently” (Shin and Moon, 2018, p. 5). This study's approach is situated within this latter perspective: it assumes that the contribution of a scientific diaspora depends on the existence of mechanisms that enable transnational cooperation among expatriate communities, scientific institutions, universities, research centers, funding agencies, and innovation ecosystems. Moreover, this approach is useful for the Venezuelan case, where scientific mobility has not necessarily resulted in permanent return, but has opened possibilities for remote collaboration, mentoring, joint research, institutional networking, and regional STI cooperation (Feld et al., 2013).

Authors such as Franco-López and Suaza-Argáez (2019) show that scientific diasporas can become strategic actors in strengthening STI ecosystems by creating academic networks, developing joint research projects, and supporting human capital development. Thus, the scientific diaspora represents a strategic resource for countries of origin, particularly in contexts of crisis or structural limitations within their science and technology systems (Pantović et al., 2026).

In the context of global development, the scientific diaspora is an essential component of knowledge management and contributes not only technical skills but also strategic links that can enhance international cooperation in the fields of science and technology. This approach aligns with the principles of the internationalization of science, which encourage the articulation of strategies and networks that extend scientific research beyond the borders of individual countries (Mentges and Costa, 2023).

Existing research has already framed the Venezuelan scientific diaspora not only as a loss of researchers, but also as a field of “linkages between members of the research community who have migrated and those who remain in the country” (Diez et al., 2020). Recent studies suggest that this diaspora constitutes an international reserve actively participating in Venezuela's reconstruction, particularly within the context of a knowledge-based society (Páez, 2024). However, interaction with Venezuela remains challenging due to the absence of clear public policies, institutional weaknesses, and limitations in the national science infrastructure (Pantović et al., 2026). These factors prevent the diaspora's integration into strategic national development projects and limit its potential contribution to strengthening Venezuela's scientific and technological system (Páez, 2024). The scientific diaspora links to Venezuela are manifested in various ways, including participation in research projects, technical consultancy, academic networking, and cooperation in innovation programmes. However, this interaction continues to face challenges linked to the absence of clear public policy, institutional weaknesses and the limitations of national science infrastructure (Villasmil, 2022).

From a science diplomacy perspective, engagement with the scientific diaspora can be framed as a knowledge-based instrument. Its value lies not only in the expertise of individual professionals, but also in the ability to mobilize scientific knowledge, professional networks, and cooperation mechanisms across institutions and borders. This perspective is consistent with Flink and Schreiterer's understanding of STI as both an issue in international relations and a tool, particularly when international scientific cooperation contributes to “capacity-building” and cross-border civil relations (Flink and Schreiterer, 2010; p. 665). It also aligns with their argument that effective science diplomacy requires the capacity to “team up different players” and make “joint priorities and objectives” feasible through appropriate organizational arrangements, funding streams, decision-making, and coordination (Flink and Schreiterer, 2010, p. 670).

The literature on engagement policy suggests that the development of science diplomacy mechanisms and networking platforms may facilitate the integration of the diaspora into the national development projects (Jasanoff, 2017). From the science diplomacy perspective, Venezuelan scientific diaspora engagement could strengthen “identity and official cooperation” while positioning diaspora members as “active nodes in the scientific ecosystem” that support knowledge circulation and regional STI cooperation (Echeverría-King et al., 2025; pp. 3–4). In this regard, international experience provides a useful reference point for designing engagement strategies that could strengthen the role of the Venezuelan diaspora in the development of national science and technology development. Comparisons with other countries in the region allow us to identify best practices and lessons learned that could be applied to Venezuela [e.g. Argentinian case according to Pombo (2025)]. These include creating digital platforms for diaspora involvement, developing international cooperation programmes and implementing public policies to recognize the importance and integrate the scientific community abroad.

Literature on science diplomacy also shows that states are increasingly interested in using science as a tool for foreign policy and development, while articulating agendas that combine research, innovation, and international relations (for example, bilateral and multilateral cooperation on global issues such as climate change, AI governance and health security). Examples on science diplomacy in non-hegemonic contexts explicitly recognized the strategic role of the scientific diaspora, noting that the scientific diaspora is strategic in the relationship between science and diplomacy and showing how a foreign-policy initiative could emerge from “a concrete proposal from the scientific-academic diaspora” (Pantović and Michelini, 2018; p. 248). This approach recognizes that scientists and their networks, including the scientific diaspora, play a role in design and implementation of cross-border policies and decision-making. (International Science Council, 2025).

In Latin America and the Caribbean, recent studies have shown that organized scientific diasporas are players in science diplomacy. Nevertheless, even if they participate in coordination processes with governments and institutions to support local scientific development, these dynamics are usually not formally integrated into national science and technology policy strategies. Therefore, there is the need for public policies that recognize and strengthen the role of diasporas as agents of development and international cooperation (Echeverría-King L et al., 2022). From a development perspective, the participation of migrant researchers may benefit the quality of local scientific output, the internationalization of STI institutions, and the governance mechanisms that shape relations with the diaspora.

Since the Venezuelan scientific diaspora operates across home–host spaces rather than simply as a population located “outside” the country, this article examines it through a transnational lens. Portes et al. (1999) frame transnationalism as an emergent field concerned with activities that take place “across multiple national borders” and with the formation of transnational communities linking immigrant groups with their countries of origin. In other words, transnationalism refers to the processes through which migrants remain connected to their homelands while engaging in economic, social, or cultural activities in their countries of residence. For scientific diasporas, these transnational connections are expressed through formal and informal associations that enable scientific cooperation, access to international resources, and the co-authorship of research, thereby enhancing global governance of science (Boric Bargetto et al., 2021). Building on this perspective, the study argues that diaspora linkage should not be understood as a rhetorical aspiration, but as an institutional and governance challenge.

The key question, therefore, is how to create governance capable of connecting dispersed scientific capacities with universities, research centers, funding agencies, and STI agendas in Venezuela and across Latin America and the Caribbean. Namely, science is no longer seen solely as a technical activity, but also as a public policy issue with social, economic, and geopolitical implications. In this context, international cooperation and coordination in STI is a central element of national strategies for development and global competitiveness (International Science Council, 2025).

According to the literature, effective coordination depends on institutional incentives, engagement policies, cooperation networks, and state support. The scientific diaspora is therefore a source of expertise that can help address national challenges through science diplomacy terms. This entity can function as an adviser in international decision-making processes and as an initiator of action, facilitating the integration of knowledge production with national priorities, institutional planning, and STI cooperation agendas (Pantović and Michelini, 2018, p. 244). Science governance could, therefore, include strategies to involve scientific diasporas and maximize their contribution to the development of their country of origin (Echeverría-King L et al., 2022).

This article advances current debates by observing the Venezuelan scientific diaspora through different conceptual frameworks: a shift from brain drain toward brain linkage, with the central issue in the Venezuelan case being not solely whether scientists return or remain abroad, but whether key stakeholders can convert diasporic identification, professional networks, and willingness to collaborate into sustained mechanisms for knowledge circulation, STI cooperation, and regional development. This approach also serves as the analytical foundation for interpreting survey and focus group results and for formulating policy recommendations to enhance diaspora governance, international scientific collaboration, and STI capacity building especially in Venezuela and in Latin America and the Caribbean region in general. Accordingly, the study does not seek to demonstrate measurable STI outcomes at scale.

## 4 Methodology

The methodological approach employed a mixed-methods, descriptive–analytical, cross-sectional design conducted between July 2025 and March 2026. This approach aimed to capture both the scope and perspectives of the Venezuelan scientific diaspora. The online questionnaire identified broader patterns, while three focus groups provided interpretive depth by examining participants' understandings of collaboration, barriers, and opportunities in practice. This strategy was deemed suitable because the research addressed both observable dimensions, such as academic profile, country of residence, links with Venezuela, and interest in collaboration, and interpretive aspects, including perceived barriers, motivations, sense of belonging, and meanings associated with transnational cooperation.

The structured online questionnaire served as the main source of quantitative data and as a qualitative input through open-ended responses. The anonymized dataset comprised 337 valid responses and 29 substantive variables. The survey collected information on sociodemographic and academic profiles, professional integration, connections with Venezuelan institutions, interest in collaboration, obstacles and motivations, expectations regarding return, diasporic belonging, participation in science, technology, and innovation activities, and perceptions of mechanisms for articulation. Methodologically, this provided a very broad empirical foundation for identifying patterns of collaboration, barriers, and potential engagement, while also enabling tangible interpretation of meanings and repertoires of action through open questions. Although the results offer significant analytical value, they are not treated as a statistically representative of the entire Venezuelan scientific diaspora.

The geographical composition of the survey sample should also be considered when interpreting the regional implications of the findings. Although respondents were distributed across 38 countries, the survey was not designed using a regionally stratified sampling framework. Instead, it was intended to generate exploratory evidence on a globally distributed and relatively engaged segment of the diaspora, whose capacities may be mobilized through transnational cooperation involving Venezuela and regional STI actors.

Interviews with members of the Venezuelan scientific diaspora were designed to complement the survey by providing collective reflection and qualitative nuance. A total of 11 participants were recruited through purposive and network-based sampling, utilizing diaspora-related academic and professional contacts, organized diaspora networks, and the authors' professional connections. The selection process aimed to capture diversity in disciplinary backgrounds (including scientists, professors, STEM, health specialists, social scientists, and leaders of diaspora-linked organizations), countries of residence (Belgium, Brazil, Colombia, Italy, Spain, and United States), gender profiles, and forms of engagement with Venezuela and regional STI ecosystems. The intent was not to achieve statistical representativeness but to ensure a broad qualitative sample. Participants were categorized into three groups.

Following data collection, the analysis was conducted sequentially and in a complementary fashion. Initially, the research team performed a quality review of the survey database, assessing completeness, identifying exact duplicates, ensuring consistency, and standardizing minor variations in short open-answer fields such as country of residence or institutional affiliation. The quantitative analysis was descriptive and exploratory, calculating absolute and relative frequencies for closed variables, analyzing multiple-response items by category recurrence, and developing bivariate cross-tabulations between key variables to identify internal patterns of association. The qualitative analysis proceeded inductively, working with open-ended survey responses and focus-group material to code recurrent themes, meanings, and narrative patterns. The qualitative material was analyzed through thematic coding, combining deductive categories derived from the research questions — such as collaboration practices, barriers, motivations, institutional trust, return, and capacity building — with inductive categories emerging from participants' narratives; coding decisions were discussed within the research team to validate interpretations and improve analytical consistency.

Finally, the study integrated both sources through methodological triangulation, complementing them. This means the survey and the focus groups were not treated as isolated blocks, but as two levels of evidence that illuminated different sides of the same phenomenon:

- The survey identified wider trends and patterns of profile, willingness to collaborate, perceived barriers, and preferred mechanisms of engagement;
- The focus groups and open-ended responses helped explain their meaning, tensions, and practical implications; material was also used to interpret the institutional experiences, and implications behind those patterns;

In other words, the survey sample is large enough to support descriptive and exploratory analysis, and the focus groups add interpretive depth. In the “Results and discussion” section, qualitative evidence is therefore presented not as a separate block, but as an interpretive layer that helps explain, nuance, or deepen the quantitative findings.

The combination of quantitative and qualitative data is well suited to the topic because diaspora engagement is not only measurable through variables such as country of residence, education level, or links with Venezuela, but also through trust, belonging, perceived barriers, and willingness to collaborate. Triangulation was used to identify relationships of confirmation, expansion, deepening, and nuance between quantitative and qualitative findings. This is important because it supports an empirically grounded interpretation of the Venezuelan scientific diaspora as a transnational actor whose contributions to knowledge circulation, collaboration, and scientific ecosystem resilience can be understood only by combining evidence with the lived experiences and strategies of diaspora members themselves. Table 1 summarizes the methodological steps taken.

**Table 1:** Methodological steps.

Component	Description of the methodological steps
Development and administration of an online questionnaire	An online questionnaire, with 29 substantive variables, opened for responses between September 2025-March 2026–337 valid records
Organization and implementation of three focus groups	Three 90-min focus groups were conducted with 3 to 4 representatives from Venezuelan scientific diaspora networks, selected to ensure disciplinary, geographic, and gender diversity
Analysis of results from both processes: online questionnaire and focus groups	Data collected from the online questionnaire analyzed using descriptive statistics, including frequencies and proportions for key variables such as: age, educational level, disciplinary area, reasons for migration, employment status, and return expectations, using in some of the cases generalized categorisations. The detailed and in-depth focus groups discussions were analyzed using thematic grouping, which allowed the identification of recurring related patterns such as: collaboration barriers, existing engagement mechanisms.

Source: own elaboration.

Because the study relied on voluntary participation and network-based dissemination, the sample may over represent diaspora members who are already embedded in academic, professional, or transnational collaboration networks and thus more inclined to express interest in engagement with Venezuela. Consequently, the findings should not be interpreted as statistically representative of the entire Venezuelan scientific diaspora, but rather as an exploratory and analytically valuable account of collaboration patterns, perceived barriers, and potential linkage mechanisms among a highly engaged segment of this population.

## 5 Results and discussion

From the 337 responses to the online questionnaire completed by members of the Venezuelan scientific diaspora, it is possible to gain insight into their potential for engagement with Venezuela and with STI ecosystems in Latin America. The sample comprises a highly qualified diaspora, predominantly in STEM fields and well-established abroad, as 83.1% reported holding a master's degree or higher and 81.9% had been living outside the country for 6 years or more. The results show a strong willingness to collaborate: 86.6% expressed interest in establishing relationships with institutions within the Venezuelan STI ecosystem, and 95.0% expressed some willingness to participate in STI activities. However, only 30.0% maintained active professional ties with institutions in Venezuela, while 40.9% indicated that they did not, although they would like to establish them. The main obstacles identified were a lack of funding, a lack of awareness of opportunities, and weak networks or contacts, while willingness to return was largely conditioned by employment, research support, and infrastructure. The qualitative analysis confirmed that the central gap lies not in a lack of interest, but in the distance between willingness and effective collaboration.

The focus groups deepen this diagnosis by highlighting the weakness of the institutional ecosystem meant to activate that potential. Participants described an active transnational scientific community that remains only weakly connected, with collaborations that often persist despite, rather than because of, formal institutional arrangements. Open-ended responses revealed recurring narratives about the absence of platforms, directories, visible information, and sustainable programs, as well as tensions between a willingness to collaborate and distrust of the institutional and political context. At the same time, the envisioned collaboration was not abstract: it was expressed in concrete repertoires of research, teaching, mentoring, knowledge transfer, advising, and innovation.

Nevertheless, the study does not assert that the Venezuelan scientific diaspora already produces measurable outcomes in STI at scale. Instead, it examines perceptions, expectations, collaborative practices, and self-reported willingness to engage as indicators of potential linkage capacity. In this sense, the evidence points to a significant reservoir of willingness, expertise, and collaborative repertoires that could be mobilized if appropriate institutional, financial, and relational mechanisms are established. This discussion has direct implications for designing public policy on STI, scientific internationalization, and international cooperation in Venezuela:

- Firstly, because countries facing skilled emigration not only require strategies to develop talent, but also instruments to maintain relationships with those already pursuing their careers in other contexts.
- Secondly, because contemporary internationalization cannot be reduced to outgoing mobility or institutional prestige, but must incorporate distributed networks, remote collaboration, mentorship, co-direction, knowledge sharing, and joint projects.
- Thirdly, because South-South cooperation offers a particularly relevant scale for countries that share funding challenges, heterogeneous scientific institutions, and development agendas linked to social, environmental, and productive problems.

In this context, funding agencies and cooperation organizations might face the challenge of designing more flexible and specific instruments for scientific diasporas, instruments that do not depend exclusively on physical return and that recognize intermediate forms of collaboration. The evidence gathered suggests that the most promising linkage mechanisms are those capable of combining funding, relational intermediation, visibility platforms, institutional hubs, and sustained cooperation schemes. The results and discussion are presented through the following six categories.

### 5.1 Demographic and academic characterization of the evaluated sample

The sample shows a relatively balanced gender composition, with a slight male majority (54.3%) compared to female participation (45.4%). This data does not, in itself, define the study's orientation, but it does confirm that the sample is not heavily biased toward either group.

A relevant analysis of the relationship between gender and area of knowledge is depicted in Table 2. This cross-tabulation allows us to observe whether the disciplinary composition of the sample differs according to gender. The estimated association was weak (Cramer's *V* = 0.15), indicating that gender does not strongly structure specialization, although relative differences between fields do appear. The main pattern shows a higher concentration of men in engineering and technology, whereas among women, the distribution is more balanced between the natural sciences and engineering. Although the difference is not significant, it suggests that the STEM composition of the sample is not homogeneous from a gender perspective. In the context of the study, this relationship provides insight into the internal configuration of the diaspora, although it does not appear to be the decisive factor in explaining the connection with Venezuela.

**Table 2:** Relation gender and area of knowledge.

Gender	Medical and health sciences	Natural sciences	Social sciences	Humanities	Engineering and technology	Other
Female	10.5	37.3	7.2	9.2	30.7	5.2
Male	6.0	35.0	6.6	2.7	46.4	3.3
Prefer not choosing	0.0	0.0	0.0	0.0	100.0	0.0

Regarding the place of residency or host country, the sample reflects a widely distributed diaspora, present in 38 countries after basic data cleansing. However, this dispersion shows clear points of concentration: Spain (19.6%), the United States (15.7%), Colombia (10.7%), and Ecuador (8.0%) account for 54.0% of the cases. This shows that the surveyed Venezuelan scientific diaspora is not concentrated in a single location, but rather distributed across Europe, North America and the Andean countries, reinforcing the need to think about the link in a transnational rather than solely bilateral framework.

An interesting cross-analysis allows us to examine whether identification with the scientific diaspora varies according to the territorial context of residence. The association was weak (Cramer's V = 0.14), suggesting a relative stability of the sense of belonging across countries.

Table 3 shows an overall trend with a predominance of total identification with the diaspora in almost all countries. This indicates that the sense of belonging does not depend heavily on place of residence, but rather remains relatively stable across diverse territorial trajectories. In analytical terms, diasporic identity appears to constitute a cross-cutting resource for articulation, although, as other cross-sections show, this identification does not automatically translate into organized networks or active cooperation.

**Table 3:** Relationship country of residence and identity as a scientific diaspora member (%).

Host country	Partly	No,I do not identify as a diaspora	Yes, totally
Alemania	7.1	7.1	85.7
Argentina	41.7	0.0	58.3
Chile	33.3	13.3	53.3
Colombia	30.6	2.8	66.7
Ecuador	18.5	3.7	77.8
España	27.3	7.6	65.2
Estados Unidos	18.9	11.3	69.8
Francia	18.2	9.1	72.7
Others	20.4	8.7	70.9

**Figure 1:**
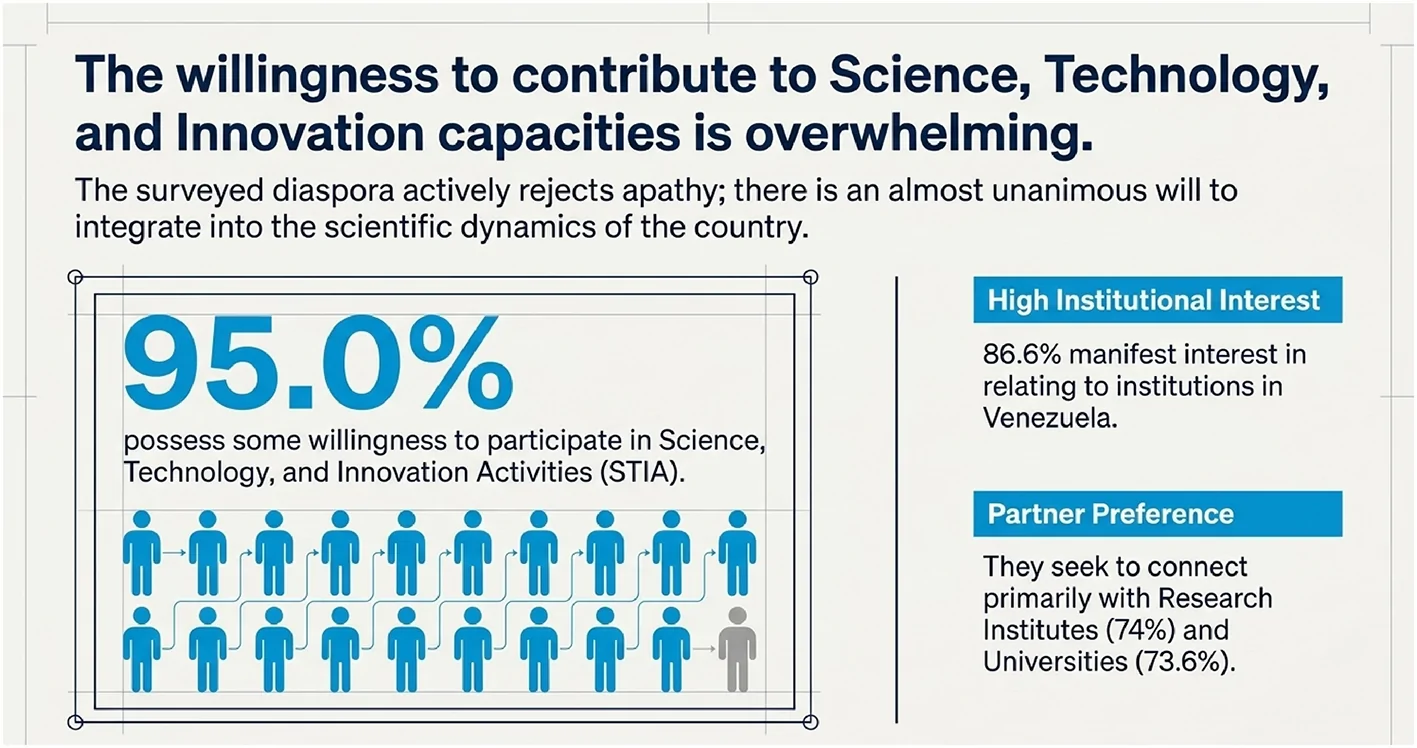
Willingness to contribute to STI—Venezuelan scientific diaspora.

The length of residence outside Venezuela confirms that these are, for the most part, established migration trajectories. A total of 42.7% reported having lived abroad for more than 10 years and 39.2% for between 6 and 10 years, meaning that 81.9% had spent at least 6 years outside the country. Figure 1 clearly shows that the survey primarily captured a medium- and long-term diaspora, which has significant implications for public policy: the relationship with this population cannot be framed solely in terms of immediate return, but also as one of sustained cooperation from abroad.

Academic attainment is one of the most consistent features of the sample. A total of 34.1% reported holding a doctorate, 27.6% a master's degree, and 20.8% having completed postdoctoral studies; overall, 83.1% held a master's degree or higher. The survey captured a highly qualified diaspora with a significant accumulation of academic capital and, therefore, strong potential for involvement in research, advanced teaching, mentoring, and specialized scientific cooperation. This educational profile is further reflected in the distribution of fields of knowledge composition heavily concentrated in STEM areas. Engineering and technology represent 39.5% of cases and natural sciences 35.9%, while medical and health sciences represent 8.0%. Overall, 75.4% of the sample belongs to engineering/technology or the natural sciences, placing the analysis within a segment that is particularly relevant to research, development, and innovation agendas.

The profile of the Venezuelan scientific diaspora is characterized by high qualifications, a concentration in STEM fields, and long careers abroad, along with a self-image of effective contributions. Quantitative data describe a consolidated sample abroad, while open-ended responses show that this consolidation does not imply disengagement. Rather, it reflects the availability of scientific, technical, and organizational skills that can be mobilized. The complementarity of these two approaches allows us to interpret the diaspora not only as emigrated human capital, but also as a distributed capacity with potential for cooperation.

On the other side, the focus groups brought together 11 highly qualified, interdisciplinary participants from biology, biotechnology, political science, computer engineering, chemical engineering, sociology, medicine, physics, microbiology, and diaspora studies. Their academic backgrounds included master's degrees, doctorates, postdoctoral training, and experience in university teaching and research. Geographically, they represented a transnational diaspora spanning the United States, Belgium, Brazil, Italy, Spain, and Colombia. The group included 4 women and 7 men.

### 5.2 Professional development and work integration

The professional placement of the sample is heterogeneous at the institutional level, but it presents a defined sectoral structure. Although open-ended responses regarding current institutions are highly dispersed and do not identify a dominant organization, classification by institution type reveals that 50.6% work in universities or research centers and 37.4% in private companies. This dual concentration suggests that the surveyed diaspora is primarily located in spaces of knowledge production and application, with the potential to shape academic and technological agendas.

Academic level and type of institution where they work is relevant because it allows us to relate academic capital and labor market integration, two central dimensions for understanding the types of cooperation that the diaspora could offer. The association was moderate (Cramer's *V* = 0.25), indicating a more structured relationship between the two variables.

Table 4 shows the concentration of PhD and postdoctoral profiles in universities and research centers is striking, while those with lower levels of education are relatively more prevalent in the private sector. This suggests that the type of professional integration of the diaspora is associated with the level of academic achievement and, therefore, with the type of contribution that can be made: more institutionalized scientific cooperation at the highest levels and potentially more applied or sector-specific links to other profiles.

**Table 4:** Relation educational attainment and institution of work (%).

Educational attaintment	Private sector	Government	Education institution	NGO	International organization	University / research center
Doctoral Degree (PhD)	18.7	2.8	0.0	4.7	2.8	71.0
Special Diploma	50.0	25.0	0.0	25.0	0.0	0.0
Bachelor	71.1	5.3	2.6	2.6	2.6	15.8
Master	56.1	8.5	0.0	2.4	3.7	29.3
Other	50.0	0.0	0.0	0.0	0.0	50.0
Postdoctoral	20.6	2.9	0.0	2.9	2.9	70.6
Undergrauduate	71.4	14.3	0.0	0.0	0.0	14.3

### 5.3 Active links with home country and commitment to collaborate

In terms of active links with Venezuela, the results show an ambivalent relationship: 30.0% maintain active professional ties with institutions in the country, 29.1% do not, and 40.9% stated that they do not currently have ties but would like to establish them. This shows that the largest group is not fully disengaged; rather, it consists of those who desire but have not yet established ties. Analytically, this suggests that the main gap is not a lack of interest, but rather a lack of effective collaboration.

Focus-group evidence shows that active links with Venezuela are already present in the form of diaspora-led university networks, remote teaching, thesis supervision, and student support. At least five participants explicitly referred to such connections, for example through “an organization… that connects graduates of Simón Bolívar University of Venezuela” (Participant 1), “teaching remotely… I'm collaborating there” (Participant 2), and being “in contact with scientific and educational activities in Venezuela… through thesis students or serving on thesis juries” (Participant 3).

Participation in projects with Venezuelan institutions reaches 44.8%, while 55.2% have not had such experience. However, this result should be interpreted alongside the strength of prior ties: among those who already have links with Venezuela, 83.2% have also participated in projects, compared to 18.4% among those without such ties. The evidence shows that cooperation exists, but it is not widespread; rather, it is concentrated among those who already have some form of prior institutional or professional connection.

This is the most important cross-tabulation in the bivariate analysis because it directly connects the existence of prior relationships with the actual activation of cooperation. As it is seen in Table 5, the association was strong (Cramer's *V* = 0.52), the highest of all the calculated cross-tabs.

**Table 5:** Relation between institutional links with Venezuela and project participation (%).

Institutional links with Venezuela	Not participation in projects	Yes, participation in projects
No	81.6	18.4
No, but I would like to	64.5	35.5
Yes	16.8	83.2

The pattern is clear: participation in projects is concentrated where prior ties already exist. Among those with professional links to Venezuela, 83.2% have also participated in projects; among those without such links, that proportion drops to 18.4%. Even the group without ties but wishing to establish them falls somewhere in between. The analytical relevance of this finding is high because it shows that cooperation depends not only on declared interest but also on the existence of active relationships that enable access, trust, coordination, and concrete opportunities for collaboration. In other words, prior ties act as mechanisms for activating scientific cooperation.

Interest in collaborating with the Venezuelan science, technology, and innovation (STI) ecosystem emerged as one of the survey's strongest findings: 86.6% of respondents expressed interest in establishing relationships with institutions within this ecosystem. This confirms that, among the sample studied, collaboration with Venezuela is a widespread expectation rather than a marginal position.

This interest also reveals specific orientations. Among those interested in collaborating, research institutes (74.0% of valid responses) and higher education institutions (73.6%) predominate, followed by companies (49.7%). Similarly, preferred activities are concentrated in joint research projects (76.2%), virtual or in-person teaching (69.2%), and technical or scientific consulting (67.9%). Consequently, the willingness to collaborate is primarily directed toward knowledge-intensive mechanisms focused on training and technology transfer, rather than peripheral or exclusively commercial approaches.

Considering the relationship between time spent abroad and interest in collaborating with Venezuela, Table 6 shows a cross-tabulation examining whether the duration of residence abroad is related to the willingness to establish institutional ties. The association was weak (Cramer's V = 0.13), but the pattern is noteworthy: interest remains high even across prolonged migration trajectories. The prevailing trend is that interest in collaborating remains high in almost all groups, although it declines among those who have been abroad for less than a year. This suggests that the initial stabilization of the migration trajectory may foster a willingness to collaborate, while prolonged residence does not, in itself, imply a disconnection from the country of origin. Analytically, this result challenges the idea that a longer stay abroad necessarily weakens the interest in maintaining ties.

**Table 6:** Relation time residing abroad and engagement with Venezuela.

Period of time residing abroad	No—interest in engaging with Venezuela	Yes, interest in engaging with Venezuela
1–5 years	10.0	90.0
6–10 years	13.6	86.4
Less than 1 year	36.4	63.6
More than 10 years	12.5	87.5

### 5.4 Motivations and obstacles for engagement

Participants from focal groups mention diverse barriers, described primarily as systemic rather than individual. They referred to the absence of institutions and formal channels for engagement, noting that “There are no institutions to make an engagement” and “things happen when you know someone who can connect you and help.” (Participant 1), that “We don't know who to talk to or where to channel proposals” (Participant 2), and that “Everything moves through contacts, not through formal channels” (Participant 5). Financial fragility was equally salient: “If you ask me what the diaspora needs: money” (Participant 9), while Participant 10 warned that “There are good ideas, but without funding they remain just intentions.” Focus groups also revealed generational exclusion and transnational frictions, as Participant 6 observed that “Many calls for proposals require impossible backgrounds for those of us who have just migrated,” and Participant 2 stressed that “Sometimes the problem isn't ability, but immigration status.”

The relation between perceived obstacles and interest in collaborating is revealing. This cross-section allows us to examine whether the interest in collaborating with Venezuela is accompanied by a lower perception of barriers or, conversely, whether both dimensions coexist. The results clearly show the latter as seen on Table 7. Even among those who express interest in collaborating, high levels of perceived obstacles persist, particularly lack of funding (73.3%) and lack of awareness of opportunities (71.6%), followed by lack of networks or contacts (56.8%) and lack of infrastructure (54.8%). Bureaucracy also maintains a significant presence (53.8%), while the perception of a lack of interest appears at a lower level (38.0%). Analytically, this pattern is especially important because it indicates that the willingness to collaborate does not automatically translate into favorable conditions for doing so. The main barrier does not appear to be a lack of interest from the diaspora, but rather the persistence of institutional and intermediary restrictions that hinder the transformation of that willingness into effective ties. In other words, the interest exists, but it coexists with an environment perceived as unfavorable for cooperation.

**Table 7:** Obstacles and interest for collaboration (%).

Obstacles	No	Yes
Bureaucracy / complex red tape	51.1	53.8
Lack of awareness of opportunities	57.8	71.6
Lack of funding	60.0	73.3
Lack of infrastructure	60.0	54.8
Lack of networking /contacts	40.0	56.8
Perception of lack of interest	40.0	38.0

In contrast, the strongest motivations are relational. Participation in international networks (70.6%) and opportunities for networking (64.1%) outweigh professional recognition (43.6%) and funding opportunities (42.1%). Collaboration is valued not only for access to resources, but also for integration into exchange networks, greater visibility, and scientific cooperation.

**Figure 2:**
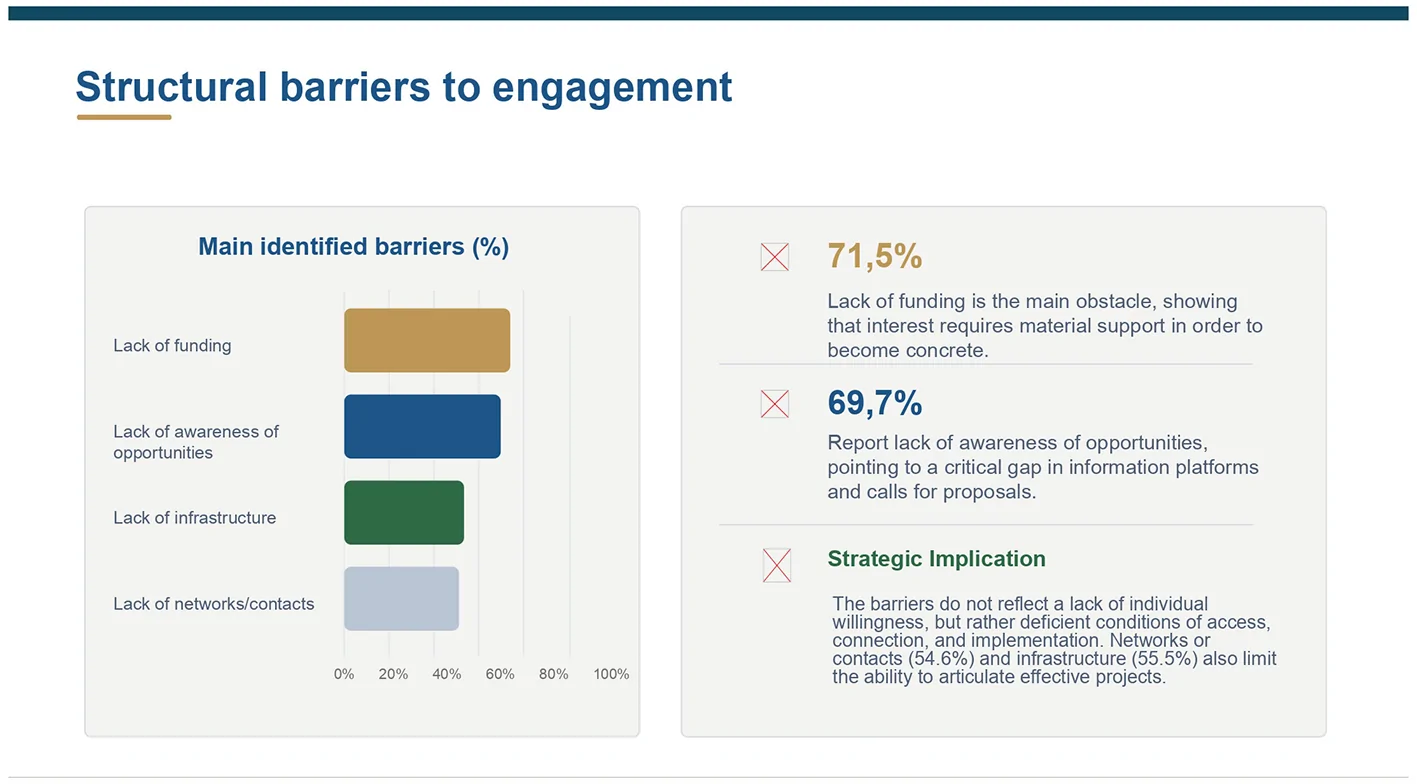
Structural barriers to engagement.

Figure 2 presents the structural barriers noted by the surveyed diaspora. For the entire sample, the most commonly cited difficulty was a lack of funding (71.5%), with limited awareness of collaboration opportunities coming next at 69.7%, followed by inadequate infrastructure at 55.5% and weak networks or contacts at 54.6%. The similarity between the first two percentages is notable and indicates that the issue stems from both a shortage of resources and limited access to information and the various institutional entry points. Therefore, collaboration with Venezuela depends mainly on identifying opportunities, setting up contact with institutions, or obtaining appropriate funding. Hence, Figure 2 supports the view that the primary constraint is not a lack of interest on the part of diaspora scientists but rather insufficient infrastructure to link dispersed scientific capacity with tangible initiatives.

The willingness to return appears to be largely conditional. Only 10.1% responded affirmatively, while 52.2% indicated that they would return depending on the conditions, and 37.7% stated that they would not consider it. Return is neither a dominant expectation nor a ruled-out option; rather, it remains a possibility contingent on specific reintegration conditions. In this context, participant 8 stated that “there are many scientists who are already well established in other countries… and they are not going to return to Venezuela, but they are still Venezuelans.”

The relationship between motivations and intention to return reveals that returning is not solely a material decision, but also a possibility linked to expectations of social and professional integration as seen on Table 8. Among those who would consider returning, the opportunity to establish contacts reaches 82.4%, a higher proportion than that observed among those who would not return (58.3%) and also higher than that of the group that responded “maybe” (64.8%). Participation in international networks is particularly relevant among those who envision a conditional return (75.6%) and remains high among those who would return (67.6%). Specific government programs also carry more weight among those who would consider returning (29.4%) than among those who would not (11.0%). Taken together, these results suggest that the willingness to return depends not only on economic or employment incentives, but also on the expectation of having networks, contacts, and institutional frameworks that make reintegration viable. Thus, the return appears less as an isolated individual decision and more as a possibility mediated by relational conditions that allow the reconstruction of scientific and professional trajectories.

**Table 8:** Motivations and willingness to return.

Motivation	No	Yes	Maybe, depending on the conditions
Opportunity to network	58.3	82.4	64.8
Financing opportunities	37.8	50.0	43.8
Participation in international networks	64.6	67.6	75.6
Specific government programs	11.0	29.4	20.5
Professional recognition	43.3	47.1	43.2

Those who would consider returning place a particularly high value on the opportunity to network, and those who respond “maybe” highlight participation in international networks. Specific government programs also carry more weight among those contemplating return than among those who would not. This suggests that the willingness to return is linked not only to material incentives but also to expectations of relational and institutional integration. Return, in this sense, appears less as an isolated individual decision than as a possibility conditioned by the existence of networks, contacts, and support systems.

Among those who would or might consider returning, the most important conditions are an attractive job offer (80.5% of valid responses), research support (65.7%), and adequate infrastructure (60.5%). Return is evaluated in terms of professional continuity and the tangible impact of scientific work, rather than as a merely symbolic or identity-related decision.

### 5.5 Diasporic identity, networking and sense of belonging

The scientific identity of the sample is distributed across several reference points. The most frequent option was identification with a global or scientific network (30.3%), followed by the scientific community in the country of residence (26.1%) and that of the country of origin (22.3%). That illustrates this plurality and suggests that the scientific affiliation of the diaspora is not limited to a single national anchor, but instead combines local, national, and transnational scales.

Even with this plurality of references, the sense of belonging to the Venezuelan scientific diaspora remains high: 69.1% consider themselves fully part of it and 23.1% partially. The evidence indicates that the notion of diaspora possesses subjective legitimacy among those surveyed, constituting an important symbolic resource for future articulation strategies. This interpretation is reinforced by Participant 11 in the focus groups, who described the diaspora as “an enormous asset” and “Venezuela's greatest international reserve.” Such a statement is analytically important because it not only confirms the persistence of collective identification among diaspora, but also reframes the diaspora from a narrative of loss to one of strategic capacity and potential national value.

This identification, however, does not automatically translate into organization. Only 11.0% reported belonging to an organized scientific diaspora network, while 89.0% indicated that they did not. This gap between identity and relational structure, points to a still fragmented organizational infrastructure.

The experience of acting as a bridge between Venezuela and the country of residence presents an intermediate situation: 49.9% have never fulfilled this role, while 32.6% have done so on some occasions and 11.6% once. Even without dense formal networks, there is a significant volume of occasional mediation that could serve as a basis for more structured connection strategies. The role of acting as a bridge between Venezuela and the country of residence emerged clearly when Participant 7 proposed a model of engagement based on events that would allow diaspora researchers to present their work and trajectories to universities and research groups in the host country. Her intervention is particularly relevant because it conceptualizes the diaspora as a bridging actor capable of connecting expertise, institutional needs, and collaborative opportunities across national contexts.

An intersection central to evaluating the function of networks as intermediary infrastructure involves also the perceived action bridging home-host country (see Table 9). The association was moderate (Cramer's *V* = 0.26), suggesting a consistent relationship between organizational membership and the ability to act as a link.

**Table 9:** Networking and perception of acting as a bridge host-home country (%).

Active networking	Never	Yes, sometimes	Yes, frequently	Just once
No	53.3	29.7	4.7	12.3
Yes	21.6	56.8	16.2	5.4

Among those belonging to organized networks, 73.0% have acted as a bridge at least once, compared to 46.7% among those not belonging to networks. Furthermore, the “yes, frequently” category is significantly higher among network members. This result suggests that networks not only express symbolic belonging but also increase the likelihood of effective scientific intermediation between Venezuela and countries of residence. For the research problem at hand, this implies that strengthening networks would not only expand the organization of the diaspora but also its concrete capacity to connect institutions, people, and opportunities for cooperation.

The open question about network membership (Q24) had a low density relative to the total sample size, which is consistent with the low percentage of people who reported belonging to an organized network. However, the 37 non-blank responses revealed a heterogeneous relational structure: academic or research-related networks predominated, followed by diaspora and mobility networks, disciplinary networks, alumni spaces, and informal connections. The emerging form of articulation is not that of a single or centralized community, but rather a fragmented relational capital, dispersed among multiple nodes and forms of membership.

Two representative expressions aptly summarize this diversity: “Colombian-Venezuelan Human Mobility Network” alludes to a diasporic and transnational articulation, while “Through friendships…” refers to less formalized ties that also function as effective channels of connection. These formulations suggest that the diaspora does not start from scratch in relational terms, but it does lack a sufficiently institutionalized infrastructure that allows these contacts to scale up to more sustained forms of cooperation.

As we can see on Table 10, the distribution of categories indicates that the existing network is not dominated by formal diaspora networks, but rather by a combination of thematic associations, mobility collectives, and informal ties. This configuration allows us to interpret the low level of institutionalization as a structural condition of the phenomenon: the diaspora already has connections, but these are neither centralized nor transformed into a visible platform for coordination. In terms of public policy, this implies that the task is not only to “create networks,” but also to recognize, map, and articulate a relational capital that already exists, albeit in a dispersed form.

**Table 10:** Related categories on network membership.

Category	Definition	Approximate recurrence	Examples
Academic/thematic research networks	Spaces for scientific collaboration organized around specific agendas, projects, or fields	20 mentions (54.1%)	“CEVALE2VE, LA-CoNGA, EL-BONGÓ”
Diaspora and mobility networks	Groups that articulate Venezuelan belonging, academic mobility, or circulation between countries	15 mentions (40.5%)	“Red Colombo Venezolana de Movilidad Humana”
Disciplinary networks / scientific societies	Professional or scientific associations linked to specific disciplines	12 mentions (32.4%)	“Sociedad Latinoamericana de Infectología Pediátrica”
Alumni and student networks	Links built from educational trajectories and institutions of origin	5 mentions (13.5%)	“AlumnUSB ”
Informal or non-institutionalized professional spaces	Friendships, consulting, or less formalized contacts	4 mentions (10.8%)	“Por relaciones de amistad…”

**Figure 3:**
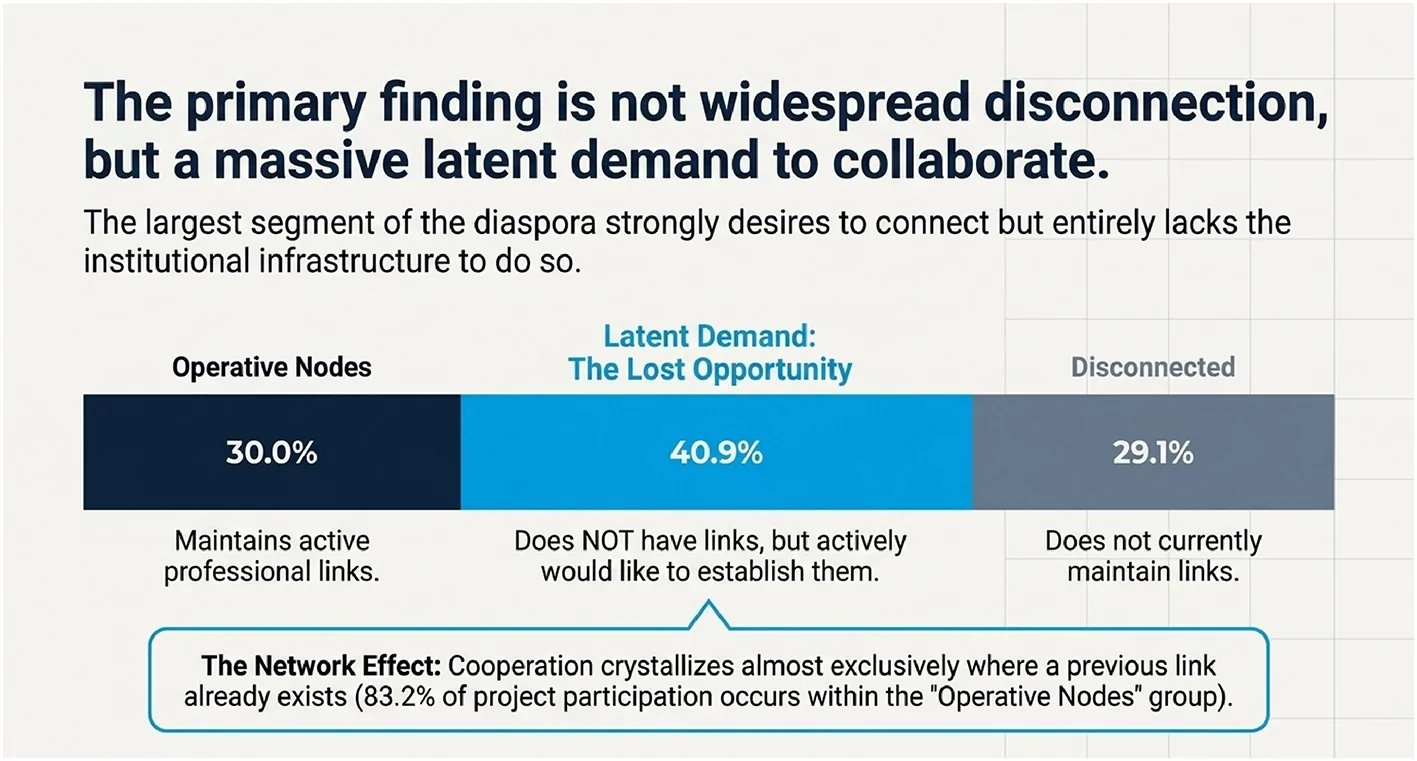
The networking effect—Venezuelan scientific diaspora.

Figure 3 visually synthesizes the central pattern discussed in this Section and Section 5.3.: the distinction between active connection, latent willingness to establish links, and current disengagement within the surveyed diaspora. The analytical significance of this distribution lies in the size of the latent-engagement group. The main challenge is therefore not simply to reconnect a disconnected diaspora, but to create institutional pathways capable of activating an already expressed willingness to collaborate. This interpretation is also consistent with the strong association between prior institutional links and project participation reported in Table 5.

### 5.6 Participation in science, technology, and innovation (STI) activities

Interest in participating in STI activities related to Venezuela is very high. A total of 55.8% responded affirmatively, depending on the conditions; 22.6% expressed strong interest; 16.6% indicated that they might participate if institutional support existed; and only a marginal proportion responded negatively. Participant 8 from focal groups stated that “everything ends up depending on personal effort.” This result confirms that the diaspora's willingness to engage in STI activities is widespread, although mediated by feasibility conditions and the lack of adequate models of engagements.

The activities for which funding would be requested are concentrated in applied research (60.8%), Research & Development activities (44.2%), and basic research (43.0%). Demand for support is primarily focused on the production and application of knowledge, consistent with the academic and disciplinary profile of the sample.

Regarding interest in STI and funding opportunities, this analysis shows that a greater willingness to participate in science, technology, and innovation activities does not translate into a generic demand for support, but rather into a relatively defined orientation toward certain types of activities. Among those who responded “Yes, very much so,” the highest priority is concentrated in applied research (73.7%), followed by R&D activities (61.8%) and basic research (53.9%). Activities such as acquiring external knowledge (48.7%) and experimental development (42.1%) also increase in this group. Among those who expressed conditional interest, the hierarchy remains, although with lower intensity, and among those who depend on institutional support, applied research also predominates (53.6%), followed by R&D (41.1%) and basic research (35.7%). This pattern suggests that interest in STI is closely associated with a vision of participation focused on the production and application of knowledge, rather than on peripheral or commercial activities. Analytically, this reinforces the idea that the surveyed diaspora conceives of its potential contribution in terms of research, development, and specialized transfer, which has direct implications for the design of financing instruments: the most relevant support would not be generic, but rather that which is capable of sustaining agendas of applied research, technological development, and scientific-technical cooperation, all the previous information is located on Table 11.

**Table 11:** Relationship between interest in STI and opportunities for which I would seek financing (%).

Activity eligible for funding	No	Yes, depending on the conditions	Yes, a lot	Maybe, if institutional funding is available
R&D activities	0.0	42.0	61.8	41.1
Marketing activities for innovation	5.9	6.4	10.5	5.4
Design activities and other creative activities	23.5	9.0	21.1	12.5
Training activities related to innovation	35.3	34.0	34.2	19.6
Innovation management and organizational improvement activities	29.4	24.5	30.3	28.6
Engineering activities	29.4	25.0	27.6	25.0
Acquisition of intangible assets	0.0	11.2	17.1	21.4
Acquisition of tangible assets	11.8	10.6	17.1	17.9
Acquisition of external knowledge	23.5	34.0	48.7	32.1
Experimental development	5.9	24.5	42.1	17.9
Applied research	23.5	61.2	73.7	53.6
Basic research	0.0	44.7	53.9	35.7
Preparation for production and distribution	17.6	9.6	25.0	12.5

The most visible pattern is that those who express greater interest in STI prioritize applied research, R&D, and basic research. They also show relatively higher values for acquiring external knowledge and experimental development. This suggests that a high interest in STI does not translate into a generic demand for support, but rather into specific preferences for activities intensive in knowledge generation and application. Consequently, the willingness to participate in STI appears to be associated with a vision of cooperation focused on research, development, and specialized transfer, rather than on peripheral activities.

Expected support from funding agencies is concentrated in flexible grants adapted to local contexts (62.6%), specific programs for diasporas and marginalized communities (57.0%), and co-financing schemes with local institutions (46.6%). The demand for resources is accompanied by a demand for institutional design: what is being requested is not only funding, but also more suitable instruments for transnational scientific cooperation.

In the focus groups, four participants explicitly referred to the role of funding agencies and support mechanisms. Participant 9 emphasized the need for “funding opportunities from multilateral organizations,” and “political mechanisms” to sustain repatriation and strengthen systematic links with the diaspora abroad. Participant 10 stressed that Venezuelans are often excluded from international calls, arguing that such calls should “open those possibilities” and include “some inclusion component” specifically for the Venezuelan diaspora. She also called for opening domestic funding and support mechanisms to enable diaspora participation. Participant 11 framed support in broader strategic terms, highlighting the potential of regional articulation and the value of large migrant communities for reconstruction and integration. Participant 8 insisted that programs “cannot be political,” “have to be institutionalized,” and should seek financing “not only nationally, but internationally,” while promoting greater policy coherence.

Figure 1 presents a summary of the overall willingness of respondents to take part in STI activities and the institutional channels which they prefer. It highlights that the main policy issue is not about generating interest but rather about establishing credible and ongoing mechanisms that will turn the widespread disposition into actual collaboration. The figure also reveals that engagement preference is mainly through institutionally rooted, knowledge-intensive participation, provided that sufficient support, coordination, and funding are available. These findings should be regarded as evidence of a great deal of potential for STI engagement.

## 6 Implications per interested stakeholders

The following implications should be read as policy-oriented recommendations derived from exploratory and self-reported evidence, not as claims that diaspora engagement has already produced measurable national reconstruction or STI development outcomes at scale.

### 6.1 Implications for public policies

The study's findings suggest that public policies aimed at the Venezuelan scientific diaspora must advance toward a flexible and sustained transnational collaboration model. The evidence shows a high willingness to collaborate, but this does not automatically translate into effective cooperation due to the lack of mechanisms for coordination, visibility, and continuity. A primary recommendation is to create a governance architecture for connection that combines active directories of scientific talent abroad, matchmaking platforms between the supply and demand for collaboration, and transparent information systems on calls for proposals, projects, institutions, and opportunities for participation. These instruments should be conceived as public goods for facilitation, not as one-off initiatives, so as to reduce the gap between declared interest and effective engagement.

Additionally, it is advisable to reduce institutional barriers that affect the viability of collaboration, particularly those associated with bureaucracy, professional recognition, equivalency of academic credentials, weak inter-institutional coordination, and limited programmatic continuity. This requires simplifying procedures, creating clear pathways for researchers abroad to connect, and recognizing modalities of remote cooperation, co-direction, participation in networks, specialized advising, and distributed collaboration. In policy terms, the priority should not be focused on “bringing back” the diaspora, but rather to enable its scientific capital to be activated from multiple locations and with varying levels of commitment. This requires sustained, tailor-made medium-term policies that address particular challenges and needs of Venezuela (and its diaspora), and integrate funding, governance, institutional hubs, and monitoring mechanisms, avoiding reliance on isolated calls for proposals or discontinuous efforts.

### 6.2 Implications for universities and research centers

Universities and research centers can play a crucial role as hubs connecting the diaspora with national and regional STI ecosystems. Based on the study's findings, it is recommended that these institutions incorporate the scientific diaspora into their active internationalization strategies, not only as a population of graduates or dispersed contacts, but as an extended academic community with potential for joint research, advanced training, mentorship, and networking. Moreover, the recommendation is that the potential of the diaspora should be acknowledged by means of using them to link the diaspora with the institutions' priorities. This will not only promote collaboration within disciplines but also help with organizational learning, institutional development, the circulation of knowledge, and the development of institutional science-diplomacy capabilities.

In practice, this means creating units or focal points for connecting with the diaspora, capable of identifying profiles, mapping career paths, activating contacts, and sustaining relationships beyond individual initiatives. Likewise, it is advisable to institutionalize programs for online teaching, mentoring of young researchers, co-supervision of undergraduate and graduate theses, participation in seminars, short-term research stays, distributed laboratories, and collaborative projects that leverage the diaspora's international reach without requiring permanent physical presence. Qualitative evidence shows that a significant portion of the envisioned contribution lies precisely in these formats of knowledge exchange and capacity building.

In parallel, universities should make more strategic use of their networks of international alumni, affiliated researchers, and regional academic partners to build consortia, expand access to funding opportunities, and transform the diaspora into a bridge for cooperation with other scientific systems in Latin America and the Caribbean. This implies moving from occasional relationships with prominent individuals to an institutional policy of engagement with transnational scientific communities. Moreover, observing the issue as a regional, universities and research centers are advised to develop capacity-building workshops to train institutional teams, researchers, students, and decision-makers in how to identify diaspora expertise, design collaborative projects that serve their particular needs, access international networks, and transform dispersed contacts into sustained mechanisms for international STI cooperation.

### 6.3 Implications for funding agencies and international cooperation organizations

For funding agencies and international cooperation organizations, the study indicates that the main challenge is not stimulating a non-existent motivation, but rather designing instruments compatible with an existing disposition, albeit one blocked by institutional and relational limitations. Consequently, it recommends developing specific calls for proposals for the scientific diaspora that recognize the transnational nature of these trajectories and allow for funding of applied research, training, networks, mentorship, knowledge transfer, and institutional strengthening. These instruments should be flexible, co-financeable, adapted to local contexts, and have administrative requirements proportional to the type of project, thus reducing barriers to access and facilitating the entry of new actors. In addition to funding projects, agencies should invest in articulation mechanisms: regional matchmaking platforms, seed funds for network building, short-term mobility programs, support for co-direction and mentorship, and distributed cooperation mechanisms that do not require mandatory return.

Given the sample's strong interest in applied research, R&D, training, and knowledge transfer, the most relevant support is that which connects scientific agendas with concrete problems and enables inter-institutional cooperation in Latin America. In this regard, regional cooperation can serve as a strategic platform for linking the diaspora with universities, research centers, companies, and public actors in different countries, expanding the scope of collaboration beyond the bilateral relationship with Venezuela.

The central recommendation for agencies is, therefore, to fund not only projects but also the infrastructure that makes cooperation possible: information, trust, intermediation, and continuity. Overall, the recommendations derived from the study point to a shift in focus. The evidence suggests that it is more productive to move from a loss-based perspective centered on brain drain to a strategy of activating transnational scientific capital, based on the logic of brain linkage. This implies recognizing that the Venezuelan scientific diaspora is not an absent resource to be recovered solely through return migration, but rather a set of existing capabilities whose contribution depends on the existence of effective mechanisms for coordination, cooperation, and governance. For international organizations, governments, universities, and funding agencies, the primary task is not to generate interest, but to build the institutional conditions to transform that interest into sustained collaboration of public value.

## 7 Conclusions

The study concludes that the Venezuelan scientific diaspora analyzed constitutes a highly qualified population, strongly integrated into STEM fields, with extensive experience abroad, and a significant capacity to contribute to STI agendas. The convergence between the descriptive results, bivariate analyses, and open narratives shows that this is not a contingent disconnected from knowledge production, but rather a system of scientific, technical, and professional capabilities already consolidated in international arenas. In this sense, Venezuelan scientific migration has undeniably contributed to the erosion of domestic research capacity, but it should be also understood as an active reserve of potential cooperation whose contribution depends on the conditions under which it can be articulated.

The integrated evidence shows a strong willingness among the surveyed participants to collaborate with stakeholders from Venezuela. The research suggests that the category of linkage should be understood as a problem of institutional design and transnational governance. It also identifies capacities and preferred forms of engagement that could potentially be mobilized in cooperation with STI institutions in Latin America and the Caribbean. However, this willingness is not expressed as an abstract or symbolic desire, but as a concrete offer of research, teaching, mentoring, consulting, technology transfer, and applied innovation. Triangulation confirms that the quantitatively observed interest acquires qualitative depth when examining the action plans proposed by participants, their sense of belonging, and their expectations for collaboration. This allows us to affirm that the diaspora not only wants to connect but also has specific and operational methods for doing so, giving the study significant programmatic value for the design of cooperation instruments.

The Venezuelan case shows that scientific diaspora engagement fails because institutions lack the mechanisms to convert willingness into collaboration. The study's main finding is the existence of a structural gap between willingness and effective collaboration. The results show that the central problem lies in the inadequacy of a connection infrastructure and governance of collaboration. Lack of funding, weak networks, the absence of linking platforms, low visibility of opportunities, and the fragility of institutional mechanisms operate together as constraints that hinder the conversion of available potential into sustained cooperation. Therefore, collaboration with diaspora is not only a technical matter of connecting researchers, but also a politically and institutionally mediated process shaped by trust, legitimacy, resources, absorptive capacity, and the feasibility of cooperation mechanisms in a complex geopolitical context. The qualitative narratives further support this interpretation by showing that participants demand not only resources but also clear rules, long-term programs, visible channels of entry, and minimum conditions of institutional trust. From this perspective, diaspora engagement should be understood as an organizational design problem and coordination between diverse actors. Additionally, it should be understood as a potential transnational knowledge actor whose power depends on the creation of institutional mechanisms for brain linkage.

Return, for its part, does not emerge as the dominant solution or the primary horizon of the relationship between diaspora and country of origin. Both quantitative results and qualitative evidence show that the possibility of returning is conditioned by structural factors, including employment, infrastructure, career continuity, professional recognition, and institutional context. Consequently, return cannot be interpreted as a binary response or an identity-based obligation, but rather as a professional and materially mediated decision. The evidence suggests the need to build forms of scientific participation that recognize already internationalized trajectories and modalities of cooperation distributed across time and space. In other words, the Venezuelan case asks for a relevant policy, adapted for its own fragile setting, that can activate scientific capabilities that are already across countries, institutions, and networks. Without such a mechanism, collaboration will continue to depend on personal contacts and isolated initiatives.

Taken together, the findings support the relevance of a transnational engagement model. The Venezuelan scientific diaspora should be viewed as a system of available capabilities whose activation depends on conditions for collaboration. Recommendations should therefore be understood in light of Venezuela's broader political and institutional context: diaspora engagement depends mainly on the existence of credible, stable, and viable mechanisms through which cooperation can be organized. For international organizations, funding agencies, universities, and public policy actors, this implies that the most promising strategies are those capable of combining flexible funding, connection platforms, relational intermediation, recognition of established career paths, remote cooperation, and sustained collaboration programs.

The Venezuelan case also has broader relevance for Latin America and the Caribbean, as it creates opportunities for South-South cooperation, regional research networks, shared training initiatives, and collaboration on common development challenges. In this context, the Venezuelan diaspora can potentially contribute not only to Venezuela's STI ecosystem, but also to broader regional scientific cooperation. The fact that many Venezuelan researchers who migrated to Latin America are younger than those in different regions, this should not be read as a hierarchy of value, but as an indication that part of this diaspora may still be developing or consolidating its academic and professional trajectories; this creates an opportunity for Latin American STI ecosystems to engage them early through research collaboration, mentoring, training, and regional network-building, turning migration into a longer-term source of knowledge circulation and capacity development.

Engaging the Venezuelan scientific diaspora is also an opportunity to expand the capacity of Latin America and the Caribbean to produce, circulate, and apply knowledge for more inclusive and resilient development. Workshops and other forms of structured engagement with the scientific diaspora can serve as tools for regional capacity building and awareness-raising, helping universities, research centers, and public decision-makers develop the skills, networks, and strategic understanding of how to strengthen STI cooperation across Latin America and the Caribbean. The contribution of this study lies in showing that cooperation with the Venezuelan scientific diaspora does not necessarily require starting from scratch; rather, the evidence points to existing willingness, expertise, and relational capacities that could be transformed into more stable, visible, and effective scientific ties if appropriate institutional, financial, and governance mechanisms are developed.

## Data Availability

The raw data supporting the conclusions of this article will be made available by the authors, without undue reservation.
